# Investigating Barriers and Facilitators to Congruence in Place of Care Among Unhoused Palliative Care Patients

**DOI:** 10.1093/geroni/igab046.1540

**Published:** 2021-12-17

**Authors:** Michael Light, Ian Johnson

**Affiliations:** 1 Harborview Medical Center, Seattle, Washington, United States; 2 University of Washington, SEATTLE, Washington, United States

## Abstract

An important quality marker for end-of-life services is congruence with patient’s preferred place of care, but this congruence in place of care is less likely for those facing structural inequalities (Grunier et al., 2007). As homelessness among older adults in the United States grows (Culhane et al., 2013), the urgency in understanding place of care in palliative care with unhoused patients grows. This presentation illustrates results from an organizational case study (Yin, 2014) of a novel homeless palliative care team and focuses on a qualitative content analysis of charts of patients over 50 receiving care both before and during the COVID-19 pandemic (n=27). Findings highlight (1) the interplay between environmental factors, psychosocial resources and constraints, and medical acuity in determining where care can take place, (2) facilitators for care in marginal settings such as emergency shelters and encampments, and (3) where opportunities for more equitable age-friendly healthcare system interventions exist.

